# Lemongrass (*Cymbopogon citratus*) supplementation improves growth performance, intestinal function and inflammation status in weaned piglets

**DOI:** 10.1016/j.aninu.2025.09.007

**Published:** 2025-11-20

**Authors:** Jing Liang, Zhenmei Zhong, Aiyang Wang, Yulong Yin, Kaibin Zheng, Xihong Zhou

**Affiliations:** a Institute of Subtropical Agriculture, Chinese Academy of Sciences, Changsha 410125, China; b College of Advanced Agricultural Sciences, University of Chinese Academy of Sciences, Beijing 101408, China; c Institute of Resources, Environment and Soil Fertilizer, Fujian Academy of Agricultural Sciences, Fuzhou 350013, China; d College of Animal Science and Technology, Hunan Agricultural University, Changsha 410128 China; e Yuelushan Laboratory, Changsha 410128 China

**Keywords:** Lemongrass;, Inflammation;, Metabolism;, Microbiota;, Piglet;

## Abstract

This study investigated the effects of dietary lemongrass (*Cymbopogon citratus*) supplementation on growth performance, intestinal morphology, and inflammation in weaned piglets. Twenty-one weaned pigs (Duroc × Landrace × Yorkshire, 21 d of age, initial body weight [IBW] = 7.70 ± 0.25 kg, *n* = 7 per group) were assigned to one of three dietary treatments: a basal diet, a basal diet supplemented with 0.1% lemongrass (LCC), or a basal diet supplemented with 0.5% lemongrass (HCC). The trial lasted for 28 d. Multi-omics approaches, including microbiomics, metabolomics, and transcriptomics, were employed to explore the underlying mechanisms. The results demonstrated that dietary lemongrass improved growth performance and intestinal morphology (*P* < 0.001). Microbiota profiling revealed that lemongrass increased the beta diversity of gut bacteria and fungi in the ileal content. Notably, dietary lemongrass enhanced the relative abundances of beneficial microbiotas such as *Lactobacillus reuteri* (*P* = 0.045), *Weissella paramesenteroides* (*P* = 0.047), and *Kazachstania slooffiae* (*P* = 0.037). In addition, lemongrass enhanced amino acid and lipid metabolic pathways in the ileum, as evidenced by changes in related metabolite contents. Transcriptomic analysis further identified the up-regulation of genes associated with nutrient metabolism and immune signaling. Correlation analysis highlighted strong associations among microbial composition, metabolite abundance, and gene expression related to nutrient metabolism. These effects were further supported by reduced levels of inflammatory cytokines in both serum and ileal tissue (*P* < 0.001), accompanied by enhanced lipase (*P* = 0.004) and trypsin (*P* = 0.002) activities. Collectively, these results indicate that dietary lemongrass improves growth performance, intestinal morphology, digestive function, inflammatory status, and microbiota composition, suggesting its potential as a promising natural alternative to antibiotics.

## 1 Introduction

Weaning is a critical stage in piglet development, during which the gut microbiota plays a pivotal role in maintaining health (Xu et al., 2024). However, the stress associated with weaning disrupts the intestinal microbial community, leading to reduced diversity (Zhou et al., 2022). Specifically, beneficial genera such as *Lactobacillus* and *Bifidobacterium* decrease in abundance, whereas opportunistic pathogens, including *Escherichia coli* and *Salmonella* proliferate. These alterations compromise gut physiology and immune regulation (Cremonesi et al., 2022; Gresse et al., 2017). In parallel, weaning triggers adaptive changes in intestinal immune function (Dong et al., 2024), including disturbances in immune cell maturation, immunoglobulin secretion, and immune signaling pathways, ultimately resulting in weakened immune defense (Ding et al., 2022).

Given the restrictions on antibiotic use in the livestock industry, alternative strategies are urgently needed to support piglets during this vulnerable period. Plant-derived bioactive compounds have attracted increasing interest due to their ability to modulate gut microbiota, enhance barrier function, and strengthen host defense against pathogens (Santhiravel et al., 2022). Lemongrass (*Cymbopogon citratus*) is rich in aldehydes, flavonoids, alkaloids, and phenolic compounds (Asaolu et al., 2009), which exhibit antioxidant, anti-inflammatory, antibacterial, and immunomodulatory properties, making it a promising substitute for antibiotics (Tiwari et al., 2019). For instance, citral, a major component of lemongrass, alleviates inflammatory responses in Caco-2 cell line (a human colon carcinoma cell line), a well-established in vitro model of the human intestinal barrier that is widely used in nutritional studies to investigate gut health and immunomodulation (Guo et al., 2022). In livestock and poultry, lemongrass supplementation has been shown to improve nutrient digestibility and rumen microbial populations in cattle (Wanapat et al., 2008), boost rumen fermentation efficiency (Wanapat et al., 2013), enhance growth performance and feed utilization in broilers (Parade et al., 2019), and reduce *E. coli* and *Salmonella* abundance in quail ceca (Alagawany et al., 2021), thereby improving immunity. These findings suggest that lemongrass could serve as a functional feed additive in animal husbandry, particularly in poultry and ruminants. Nevertheless, its effects and mechanisms of action in weaned piglets remain unexplored.

Therefore, the objective of this study was to evaluate the effects of dietary lemongrass supplementation on growth performance, intestinal function, and microbiota composition in weaned piglets, and to elucidate the key underlying mechanisms. To achieve this, gas chromatography-mass spectrometry (GC–MS), microbial sequencing, metabolomics, and transcriptomics were applied to comprehensively elucidate the underlying mechanisms. Collectively, this research highlights lemongrass as a promising natural alternative to antibiotics in pig production and contributes to advancing the sustainable development of the swine industry.

## 2 Methods and materials

### 2.1 Animal ethics statement

The animal trial was approved by the Protocol Management and Review Committee of the Institute of Subtropical Agriculture (Approval No. 20230923) and was strictly conducted in accordance with the Animal Care guidelines of the Institute (Changsha, China).

### 2.2 Animal experiments and sample collection

A total of 21 healthy piglets (Duroc × Landrace × Yorkshire, initial body weight [IBW] = 7.70 ± 0.25 kg) weaned at 21 d of age were randomly assigned to three groups (*n* = 7 per group): a basal diet (CON), a basal diet supplemented with 0.1% *C*. *citratus* (LCC), or a basal diet supplemented with 0.5% *C*. *citratus* (HCC). Lemongrass powder replaced an equivalent amount of zeolite powder in the basal diet. The basal diet met the nutritional requirements outlined by the NRC (2012). The ingredient composition and nutrient levels of the basal diet are presented in Table 1. The nutrient profile of lemongrass powder is presented in Table 2, and its major chemical constituents is presented in Table S1.

**Table 1:** Ingredients and nutrients of the basal diet (%, as-fed basis).

Ingredients	Content	Nutrients^3^	Content
Corn (7.8% CP)	52.00	CP	18.41
Broken rice	11.00	Ether extract	3.15
Soybean meal (43% CP)	16.00	Crude fiber	3.71
Extruded soybean	6.00	DM	91.75
Fish meal	3.00	Ash	5.09
Whey powder	5.00	Neutral detergent fiber	7.71
Soybean oil	1.00	Acid detergent fiber	4.17
Fat powder	1.00	Total phosphorus	0.63
Calcium hydrogen phosphate dihydrate (CaHPO_4_·2H_2_O)	1.30	Available phosphorus	0.41
Mountain flour	0.80	Calcium	0.81
NaCl	0.30	Lysine	1.21
L-Lysine hydrochloride	0.50	Methionine	0.37
DL-Methionine	0.10	Threonine	0.73
L-Threonine	0.20	Tryptophan	0.21
L-Tryptophan	0.06	Organic matter	86.66
Multi-vitamins^1^	0.03	Gross energy, MJ/kg	15.99
Multi-minerals^2^	0.10	Net energy, MJ/kg	10.88
Zeolite powder	1.61	Digestible energy, MJ/kg	14.73
Total	100.00		

1 Provided for per kilogram of diet: vitamin A, 9750 IU; vitamin D_3_, 3000 IU; vitamin E, 24 mg; vitamin K_3_, 3 mg; vitamin B_1_, 3 mg; vitamin B_2_, 7.5 mg; vitamin B_6_, 4.5 mg; vitamin B_12_, 0.03 mg; nicotinamide, 36 mg; D-pantothenic acid, 21 mg; folic acid, 1.5 mg; D-biotin, 0.15 mg.

2 Provided for per kilogram of diet: Cu_2_(OH)_3_Cl, 12.5 mg; ZnSO_4_, 70 mg; FeSO_4_, 115 mg; MnSO_4_, 27.5 mg; Ca(IO_3_)_2_, 0.5 mg; Na_2_SeO_3_, 0.35 mg.

3 Crude protein, ether extract, crude fiber, dry matter, gross energy was actually measured, and others were calculated.

**Table 2:** Analyzed nutrition level of lemongrass powder (%).

Composition	Lemongrass powder
Crude fibre	29.16
Crude fat	10.21
Crude protein	8.21
Dry matter	96.73
Gross energy, MJ/kg	18.50

During the 28-d experimental period, each piglet was individually housed with ad libitum access to feed and water. Daily feed intake and body weight were monitored throughout the study, with measurements taken at the initial and final time points. At the end of the experiment, the piglets were fasted for 12 h, and venous blood samples were collected. Serum was obtained by centrifugation at 3000 × *g* for 10 min at 4 °C and stored at −80 °C for further analysis. Piglets were then euthanized, and digesta and tissue samples from different intestinal segments were collected, snap-frozen in liquid nitrogen, and stored at −80 °C until analysis.

### 2.3 Serum biochemistry assay

Serum biochemistry parameters, including total protein (TP; Cat. No. A045-3-2), blood urea nitrogen (BUN; Cat. No. C013-2-1), triglycerides (TG; Cat. No. F001-1-1), and cholesterol (CHOL; Cat. No. A111-1-1), were analyzed using assay kits (Nanjing Jian Cheng Bioengineering Institute, Nanjing, Jiangsu, China) with an automated analyzer (cobas c311, Roche Life Science, Basel, Switzerland) according to the manufacturer’s procedures.

### 2.4 Intestinal morphological analysis

Ileal tissues were fixed in 4% paraformaldehyde, embedded in paraffin, and sectioned to 5 μm thickness. Sections were dewaxed in xylene, rehydrated, and stained with hematoxylin and eosin (H&E; Cat. No. C0105M, Beyotime Biotechnology Co., Ltd., Shanghai, China). Representative images were captured using SlideViewer software (3DHISTECH, Budapest, Hungary).

### 2.5 Immunofluorescence staining

Ileal sections underwent dewaxing and rehydration as described above. Antigen retrieval was performed in ethylenediaminetetraacetic acid buffer (pH 9.0) at 95 °C for 20 min. After washing with phosphate-buffered saline, endogenous peroxidase activity was blocked with 3% H_2_O_2_, and non-specific binding was reduced by incubation with normal goat serum. Sections were incubated overnight at 4 °C with an anti-claudin primary antibody (Cat. No. GB152543, 1:1000; Servicebio Technology Co., Ltd., Wuhan, Hubei, China), followed by a horseradish peroxidase-polymer secondary antibody (Cat. No. GB23303, Servicebio Technology Co., Ltd., Wuhan, Hubei, China). Fluorescence labeling was performed using a tyramide signal amplification kit (TYR-570; YOBIBIO Co., Ltd., Shanghai, China) for 10 min, and nuclei were counterstained with 4’,6-diamidino-2-phenylindole (DAPI; Cat. No. C1006, Beyotime Biotechnology Co., Ltd., Shanghai, China). The relative fluorescence intensity was quantified as the ratio of total fluorescence intensity to the selected tissue area using ImageJ software (v 1.54g, National Institutes of Health, Bethesda, MD, USA).

### 2.6 Nutritional analysis of the basal diet and lemongrass powder

Prior to nutrient analysis, the basal diet and lemongrass powder were sealed in polyethylene bags and stored at −20 °C. The content of DM, ether extract, CP, and ash in the samples was determined following standard procedures of the Association of Official Analytical Chemists (AOAC, 2006).

Specifically, DM was measured by drying 2.0 g of sample at 105 ± 2 °C to constant weight (method 930.15); ether extract was analyzed using Soxhlet extraction with petroleum ether (30-60 °C) for 6 h (method 920.39); CP was determined by the Kjeldahl method, with nitrogen content converted to protein using a factor of 6.25 (method 984.13); and ash was quantified by incinerating 2.0 g of sample at 550 ± 25 °C for 6 h (method 942.05). Crude fiber (CF) was determined using fiber bags in an ANKOM A200i Fiber Analyzer (ANKOM Technology Corp., Macedon, NY, USA), following the manufacturer's instructions and previously described protocols (Van Soest et al., 1991). Gross energy was measured using an automatic oxygen bomb calorimeter (HXR-6000, Changsha Huaxing Energy Technology Co., Ltd., Changsha, China) according to GB/T 45104-2024 (China National Standard, 2024). Nutrient values, including neutral detergent fiber, acid detergent fiber, total phosphorus, available phosphorus, calcium, lysine, methionine, threonine, tryptophan, net energy, and digestible energy, were calculated based on the China Feed Database (2023).

### 2.7 GC–MS analysis of lemongrass extract

Fresh lemongrass (200 g) was mixed with 600 mL of water containing NaCl (final concentration: 9%, wt/vol), soaked in a distillation reflux apparatus for 12 h, and distilled for 3 h to obtain the extract. The chemical composition of lemongrass extract was analyzed by GC–MS. Separation was performed on an Agilent 19091S-433 HP-5MS column (30 m × 250 μm × 0.25 μm; Agilent Technologies Inc., Santa Clara, CA, USA). A 1-μL aliquot was injected with a split ratio of 50:1. The oven temperature program was the following: initial 50 °C for 5 min, increased to 100 °C at 10 °C/min (no hold), then ramped to 300 °C at 30 °C/min and held for 4 min. The mass spectrometer was operated in scan mode with a solvent delay of 0 min, a scan range of 20 to 800 m/z, and a threshold of 100. Lemongrass essential oil was extracted using the distillation apparatus (JC-ZL500, Qingdao Jingcheng Instrument Co., Ltd., Qingdao, China) following the standard protocol and diluted to a suitable concentration for GC–MS analysis.

### 2.8 Ileal microbiota profiling

Microbial DNA was extracted from ileal digesta using the E.Z.N.A. Soil DNA Kit (Cat. No. D5625–00; Omega Bio-tek, Inc., Norcross, GA, USA). The bacterial 16S rRNA gene and fungal Internal Transcribed Spacer (ITS) region were amplified by PCR and sequenced on the Illumina NextSeq 2000 platform (Illumina Inc., San Diego, CA, USA) by Majorbio Bio-Pharm Technology Co., Ltd. (Shanghai, China). Raw Fast Quality Score (FASTQ) files were quality-filtered and merged using Fast Length Adjustment of Short Reads (FLASH). Alpha diversity indices (abundance-coverage estimator [ACE], Chao1, Shannon, Simpson) were calculated, while beta diversity was assessed by Principal Coordinates Analysis (PCoA). Differences in microbial communities were identified using Linear Discriminant Analysis Effect Size (LEfSe).

### 2.9 Transcriptome sequencing

Total RNA was extracted from 100 mg of ileal tissue using TRIzol reagent (Cat. No. 15596026, Thermo Fisher Scientific Inc., Waltham, MA, USA). Briefly, powdered tissue was lysed in 1 mL pre-chilled TRIzol, incubated at room temperature for 5 min, and mixed with 200 μL chloroform. After centrifugation (13,000 × *g*, 15 min, 4 °C), the aqueous phase was collected, and RNA was precipitated with isopropanol. The pellet was washed with 75% ethanol, air-dried, and resuspended in 50 μL diethyl pyrocarbonate-treated water. RNA concentration and integrity were evaluated using the 5300 Bioanalyzer (Agilent Technologies Inc., Santa Clara, CA, USA) and ND-2000 (Thermo Fisher Scientific Inc., Waltham, MA, USA). Only samples meeting the criteria (total RNA ≥ 1 μg, concentration ≥ 30 ng/μL, RNA quality number > 6.5, optical density [OD] 260/280 = 1.8–2.2, OD 260/230 ≥ 2.0) were used for subsequent analysis.

Transcriptome libraries were prepared using the Illumina Stranded mRNA Prep, Ligation protocol and sequenced on the NovaSeq X Plus platform (PE150; Illumina Inc., San Diego, CA, USA). Raw reads were processed with fastp (v0.19.5) to remove adapters, low-quality sequences (quality threshold < 20, >10% N bases, or length < 20 bp). Clean reads were aligned to the reference genome using HISAT2 (v2.1.0), assembled with StringTie (v2.1.2), and quantified with RSEM (v1.3.3). Differentially expressed genes (DEGs) were defined as |log_2_ fold change| ≥ 1 and false discovery rate (FDR) < 0.05. Functional annotation was performed using Gene Ontology (GO) and Kyoto Encyclopedia of Genes and Genomes (KEGG) analyses. Integrated transcriptome-metabolome analysis was conducted on the Majorbio Cloud platform.

### 2.10 Metabolomics analysis

Ileal contents (50 mg) were extracted with 400 μL methanol:water (4:1, vol/vol) containing 0.02 mg/mL L-2-chlorophenylalanine. The mixture was kept at −10 °C, homogenized with a Wonbio-96c crusher (50 Hz, 6 min), sonicated (40 kHz, 30 min, 5 °C), incubated at −20 °C for 30 min for protein precipitation, and centrifuged (13,000 × *g*, 4 °C, 15 min). The resulting supernatant was subjected to LC-MS/MS analysis. A pooled quality control (QC) sample, generated by mixing aliquots from all individual samples, was analyzed at regular intervals to monitor system stability.

Metabolomic profiling was performed using an Ultra-High-Performance Liquid Chromatography (UHPLC) system coupled to a Q Exactive HF-X system (Thermo Fisher Scientific Inc., Waltham, MA, USA) equipped with an HSS T3 column (Waters Corp., Milford, MA, USA). The mobile phases consisted of 0.1% formic acid in water:acetonitrile (95:5, vol/vol) (solvent A) and 0.1% formic acid in acetonitrile:isopropanol:water (47.5:47.5:5, vol/vol) (solvent B), with gradient elution (0-10 min). Mass spectrometry analysis was performed using data-dependent acquisition (DDA) in both positive and negative electrospray ionization (ESI) modes over an m/z range of 70 to 1050. Raw data were processed using Progenesis QI (Waters Corp., Milford, MA, USA). Metabolite identification was performed against Metlin (https://metlin.scripps.edu/) and the Majorbio Database. Processed data were analyzed on the Majorbio Cloud platform, including feature filtering (≥80 % presence across samples), missing value imputation, sum normalization, QC-based relative standard deviation (relative standard deviation < 30 %) filtering, and log_10_ transformation. Differential metabolites were defined as those with variable importance in projection (VIP) > 1 from orthogonal partial least squares discriminant analysis (OPLS-DA) and *P* < 0.05 by Student's *t*-test. Multiple group comparisons and KEGG pathway enrichment analyses were further conducted using the Majorbio Cloud platform.

### 2.11 Determination of inflammatory cytokines and digestive enzymes

Enzyme-linked immunosorbent assay (ELISA) kits (Nanjing Boyan Biological Technology Co., Ltd., Nanjing, Jiangsu, China) were used to measure serum and ileal levels of tumor necrosis factor-alpha (TNF-α; Cat. No. BYHS500565), interleukin-1 beta (IL-1β; Cat. No. BYHS500664), interferon-gamma (IFN-γ; Cat. No. BYHS500562), IL-6 (Cat. No. BYHS500639), fatty acid transport protein (FATP) (Cat. No. BY-EM222948), and fatty acid binding protein (FABP) (Cat. No. SP21537, Wuhan Saipei Biotechnology Co., Ltd., Wuhan, Hubei, China), as well as the activities of lipase (Cat. No. BC2340, Beijing Solarbio Science & Technology Co., Ltd., Beijing, China) and trypsin (Cat. No. P0324S, Beyotime Biotechnology Co., Ltd., Shanghai, China), according to the instructions of manufacturer.

### 2.12 Statistical analysis

All statistical analyses were performed using SPSS Statistics 28 (SPSS Inc., Chicago, IL, USA). Student's *t*-test was used to compare differences between two groups, while one-way ANOVA was applied for comparisons among three groups, with linear and quadratic effects also tested. When significant differences were detected, Duncan's multiple range test was used for post hoc analysis. Statistical significance was set at *P* < 0.05. A mixed linear model was applied as follows:

$$Y_{ij}=\mu +X_{i}+{ɛ}_{ij,}$$

where *Y*_*ij*_ is the dependent variable; *μ* is the overall mean; *X*_*i*_ is the fixed effect of treatment; and *ɛ*_*ij*_ is the random error.

## 3 Results

### 3.1 Dietary lemongrass improves growth performance, intestinal morphology, and barrier function in piglets

**Fig. 1:**
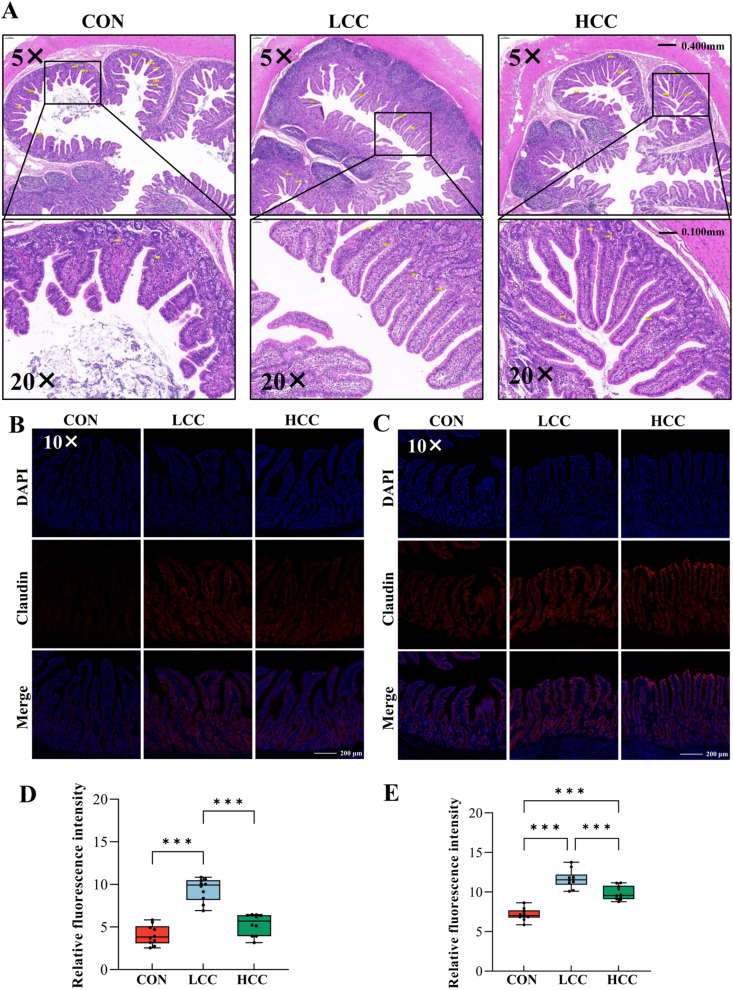
The effects of lemongrass on ileal morphology and claudin expression. Dietary treatments were as follows: CON (basal diet), LCC (basal diet + 0.1% lemongrass), and HCC (basal diet + 0.5% lemongrass). (A) Lemongrass affects ileal morphology in weaned piglets. (B and C) The expression of claudin in the jejunum and ileum. DAPI = 4′,6-diamidino-2-phenylindole. (D and E) The relative fluorescence intensity of the jejunum and ileum. Data are shown as mean ± SEM, *n* = 7 per group, ∗∗∗*P* < 0.001.

The study first evaluated the effects of dietary lemongrass supplementation on growth performance, serum biochemical parameters, and intestinal morphology. Piglets in both the LCC and HCC groups showed increased final body weight (FBW) (*P* < 0.001), ADFI (*P* = 0.002), and ADG (*P* < 0.001), accompanied by a reduced F:G ratio (*P* = 0.002), compared with the control group (Table 3). No significant differences in TP content were observed among groups (*P* = 0.652, Table S2). High-level lemongrass supplementation elevated serum BUN (*P* = 0.043) and CHOL (*P* = 0.037) concentrations, whereas no such effects were observed with the low-level treatment (Table S2). In addition, serum TG levels were higher in both the LCC and HCC groups relative to the control (*P* = 0.015, Table S2). Histological analysis of the ileum revealed improved intestinal morphology in piglets supplemented with lemongrass (Fig. 1A). Moreover, lemongrass supplementation upregulated claudin expression in both the jejunum (*P* < 0.001) and ileum (*P* < 0.001), with the effect being more pronounced at the low supplementation level (Fig. 1B–E).

**Table 3:** Effects of lemongrass on growth performance of weaned piglets.

Item	Groups^1^			SEM	*P*-value		
	CON	LCC	HCC		Treatment	Linear	Quadratic
IBW, kg	7.77	7.66	7.67	0.459	0.983	0.879	0.920
FBW, kg	16.12^b^	22.72^a^	20.65^a^	0.931	<0.001	0.003	0.001
ADG, g	298.21^b^	537.76^a^	463.52^a^	21.369	<0.001	<0.001	<0.001
ADFI, g	583.32^b^	835.61^a^	729.03^a^	41.232	0.002	0.022	0.002
F:G ratio	1.98^a^	1.56^b^	1.58^b^	0.080	0.002	0.002	0.035

SEM = standard error of the mean; IBW = initial body weight; FBW = final body weight; ADFI = average daily feed intake; ADG = average daily gain; F:G ratio = feed-to-gain ratio.

Means with different superscripts within the same row differ significantly (*P* < 0.05).

1 CON, the basal diet group; LCC, 0.1% lemongrass supplementation in the basal diet group; HCC, 0.5% lemongrass supplementation in the basal diet group.

### 3.2 Dietary lemongrass alters microbial composition of piglets

**Fig. 2:**
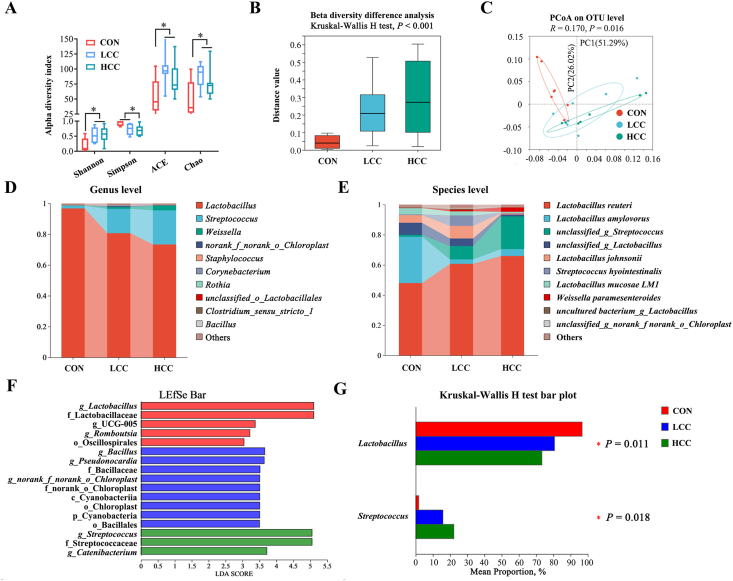
Lemongrass regulates ileal bacteria composition in weaned piglets. Dietary treatments were as follows: CON (basal diet), LCC (basal diet + 0.1% lemongrass), and HCC (basal diet + 0.5% lemongrass). (A) Shannon, Simpson, abundance-coverage estimator (ACE) and Chao indices. (B) Beta diversity at the genus level. (C) Principal Coordinates Analysis (PCoA) results at Operational Taxonomic Unit (OTU) level. (D - E) The relative abundance of microbes at the genus level and the species level. (F) Linear Discriminant Analysis Effect Size (LEfSe) analysis. (G) Kruskal–Wallis H test. Data are shown as mean ± SEM, *n* = 7 per group. ∗*P* < 0.05.

To determine whether lemongrass modulates the gut microbiota, 16S rRNA and ITS sequencing of ileal chyme was performed. Based on 16S rRNA sequencing, the LCC and HCC groups exhibited higher Shannon (*P* = 0.016), ACE (*P* = 0.007), and Chao (*P* = 0.007) indices, while the Simpson (*P* = 0.008) index was lower compared with the control group (Fig. 2A), indicating enhanced alpha diversity. The Kruskal–Wallis H test further revealed differences in beta diversity at the genus level (*P* < 0.001) (Fig. 2B). PCoA analysis confirmed distinct clustering between the control and lemongrass-supplemented groups (Fig. 2C).

At the genus level, the dominant taxa included *Lactobacillus*, *Streptococcus*, and *Weissella* (Fig. 2D). At the species level, LCC and HCC supplementation increased the relative abundances of *Lactobacillus reuteri* (*P* = 0.045) and *Weissella paramesenteroides* (*P* = 0.047) (Fig. 2E). LEfSe analysis identified 17 significantly different taxa across treatments: five enriched in the control, nine in the LCC group, and four in the HCC group (Fig. 2F). At the genus level, *Lactobacillus*, UCG-005, and *Romboutsia* were enriched in the control group; *Bacillus* and *Pseudonocardia* were enriched in the LCC group; while *Streptococcus* and *Catenibacterium* predominated in the HCC group. The Kruskal–Wallis H test further confirmed that lemongrass supplementation increased *Streptococcus* abundance (*P* = 0.018) while decreasing *Lactobacillus* (*P* = 0.011) (Fig. 2G).

**Fig. 3:**
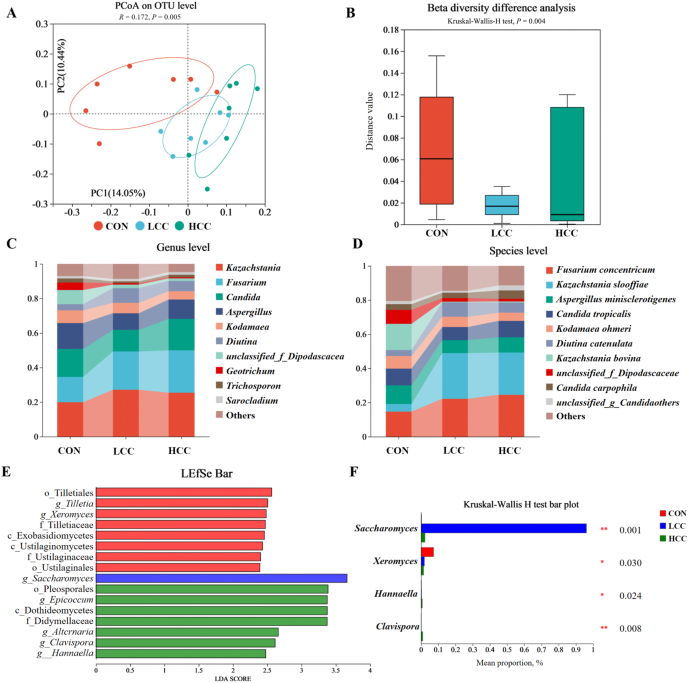
Lemongrass regulates ileal fungi composition in weaned piglets. Dietary treatments were as follows: CON (basal diet), LCC (basal diet + 0.1% lemongrass), and HCC (basal diet + 0.5% lemongrass). (A) Principal Coordinates Analysis (PCoA) results at the Operational Taxonomic Unit (OTU) level. (B) Beta diversity at the genus level. (C-D) The relative abundance of microbes at genus level and the species level. (E) Linear Discriminant Analysis Effect Size (LEfSe) analysis. (F) Kruskal–Wallis H test. Data are shown as mean ± SEM, *n* = 7 per group. ∗*P* < 0.05, ∗∗*P* < 0.01.

Internal Transcribed Spacer sequencing revealed that fungal community composition also shifted in response to lemongrass. PCoA indicated differences in fungal composition between groups (*P* = 0.005) (Fig. 3A). At the phylum level, beta diversity was reduced in the LCC and HCC groups (*P* = 0.004) (Fig. 3B). The dominant genera included *Kazachstania*, *Fusarium*, and *Candida* (Fig. 3C). At the species level, *Fusarium coacentricum* and *Kazachstania slooffiae* were enriched in the LCC and HCC groups (Fig. 3D). LEfSe analysis showed that *Saccharomyces* was enriched in the LCC group, while *Alternaria*, *Clavispora*, and *Hannaella* characterized the HCC group (Fig. 3E). Kruskal–Wallis H test revealed that beneficial fungi such as *Saccharomyces* (*P* = 0.001), *Hannaella* (*P* = 0.030), and *Clavispora* (*P* = 0.024) increased following lemongrass supplementation, with *Saccharomyces* showing the greatest increase in the LCC group. In contrast, *Xeromyces* abundance decreased in both LCC and HCC groups (*P* = 0.008) (Fig. 3F).

### 3.3 Dietary lemongrass affects metabolite profiling in the ileum of piglets

**Fig. 4:**
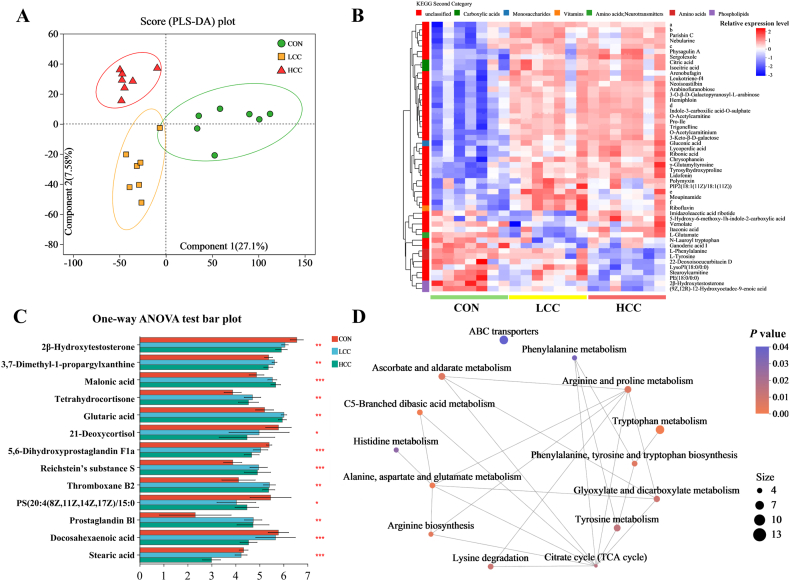
Lemongrass affects ileal metabolite composition in weaned piglets. Dietary treatments were as follows: CON (basal diet), LCC (basal diet + 0.1% lemongrass), and HCC (basal diet + 0.5% lemongrass). (A) Partial Least Squares Discriminant Analysis (PLS-DA) plot. (B) Heatmap of top 50 differential metabolites. (a to f) The chemical structure diagrams of these compounds are the following: a.  b.  c. d.  e. f. (C) Differential metabolites related to lipid mediator metabolism and fatty acid metabolism. (D) Kyoto Encyclopedia of Genes and Genomes (KEGG) pathway enrichment network analysis. Data are shown as mean ± SEM, *n =* 7 per group. ∗*P <* 0.05, ∗∗*P <* 0.01, ∗∗∗*P* < 0.001.

Metabolomic analysis of ileal chyme showed that lemongrass supplementation altered metabolite profiles, as indicated by PLS-DA (Fig. 4A). A heatmap of the top 50 differential metabolites highlighted distinct clustering among groups (Fig. 4B). KEGG annotation revealed that most differential metabolites were related to lipids and biologically active compounds. Lipid-related metabolites were mainly fatty acids and their conjugates, flavonoids, and isoprenoids (Fig. S1A), while biologically active compounds included carboxylic acids, monosaccharides, vitamins, and amino acids (Fig. S1B). One-way ANOVA identified increases in several representative metabolites, including 3,7-dimethyl-1-propargylxanthine (*P* = 0.007), tetrahydrocortisone (*P* = 0.003), malonic acid (*P* < 0.001), Reichstein's substance s (*P* < 0.001), thromboxane B2 (*P* = 0.003), and prostaglandin B1 (*P* = 0.008) (Fig. 4C). KEGG enrichment analysis revealed that differential metabolites were primarily involved in lipid metabolism, amino acid metabolism, and ATP-binding cassette (ABC) transporter pathways (Fig. 4D).

**Fig. 5:**
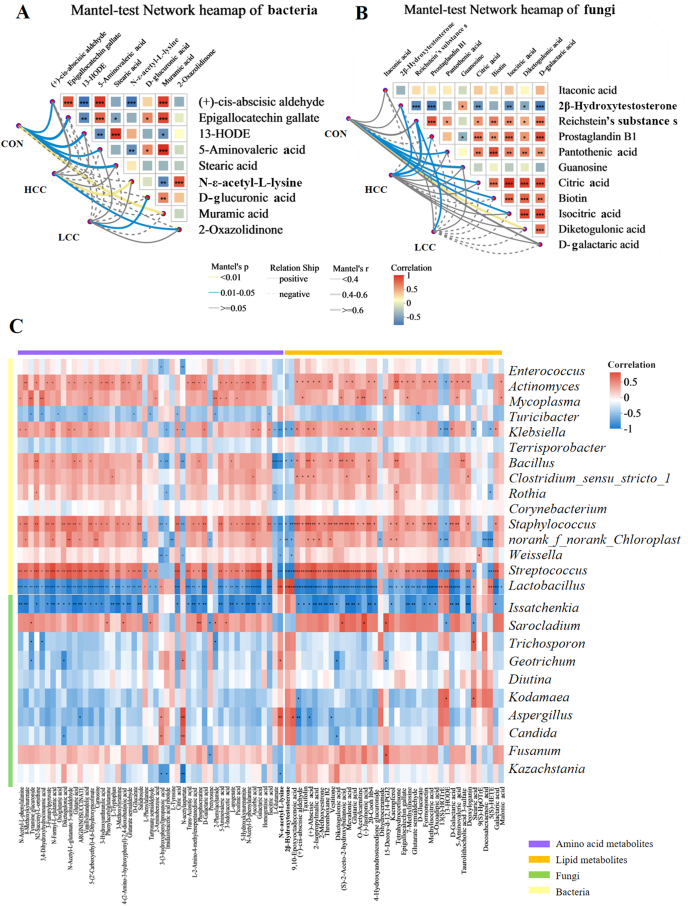
The association between microbiotas and metabolites. Dietary treatments were as follows: CON (basal diet), LCC (basal diet + 0.1% lemongrass), and HCC (basal diet + 0.5% lemongrass). (A) Mantel-test network for bacteria and metabolites. (B) Mantel-test network for fungi and metabolites. (C) Correlation analysis of ileal microbiota (green: fungi, yellow: bacteria) and metabolites (purple: amino acid metabolites, orange: lipid metabolites). 13-HODE = 13(S)-Hydroxy-[12,13-3H] octadecadienoic acid. ∗*P* < 0.05, ∗∗*P* < 0.01, ∗∗∗*P* < 0.001.

Correlation analysis between metabolites and microbial taxa identified 40 lipid-related and 50 amino acid-related metabolites associated with specific microbial communities (Fig. 5A and B). Among fungi, *Issatchenkia* exhibited the strongest associations, whereas *Streptococcus*, *Lactobacillus*, *Staphylococcus*, and *Actinomyces* were the most strongly correlated bacterial taxa. *Klebsiella*, *norank_f_norank_o_Chloroplast*, and *Bacillus* were correlated with approximately half of the metabolites. Mantel test revealed that bacterial genera were significantly associated with N-ε-acetyl-L-lysine, D-glucuronic acid, and 2-oxazolidinone, whereas fungal genera correlated with 2β-hydroxytestosterone and Reichstein's substances (Fig. 5C).

### 3.4 Dietary lemongrass modulates transcriptome profiling in the ileum of piglets

**Fig. 6:**
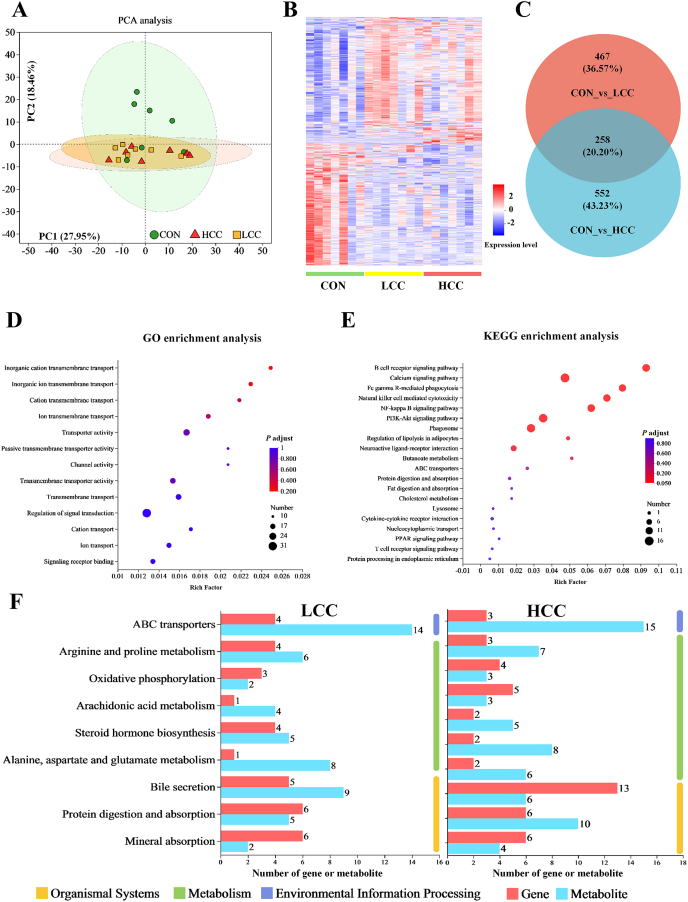
Lemongrass powder alters gene expression in weaned piglets. Dietary treatments were as follows: CON (basal diet), LCC (basal diet + 0.1% lemongrass), and HCC (basal diet + 0.5% lemongrass). (A) Principal component analysis (PCA) results. (B) Heatmap of differentially expressed genes (DEGs). (C) Venn plot of DEGs. (D) Gene Ontology (GO) enrichment analysis plot. (E) KEGG enrichment analysis plot. (F) Correlation analysis of KEGG pathway enrichment for DEGs and differential metabolites, ABC transporter = ATP-binding cassette transporter.

Transcriptomic profiling of ileal tissue demonstrated significant alterations in gene expression following lemongrass supplementation. Principal component analysis (PCA) and hierarchical clustering showed clear group separation (Fig. 6A and B). Differential expression analysis revealed 177 upregulated and 548 downregulated genes in the LCC group, and 327 upregulated and 483 downregulated genes in the HCC group, relative to the control (Fig. S2A–B). A total of 258 DEGs were shared between the two treatment groups (Fig. 6C). GO enrichment indicated that these DEGs were primarily involved in signal transduction, transporter activity, and transmembrane transport (Fig. 6D). KEGG pathway enrichment showed involvement in B and T cell receptor signaling, lipolysis regulation, cytokine–cytokine receptor interaction, fat digestion and absorption, and nuclear factor kappa-B (NF-κB) signaling (Fig. 6E). Integration of transcriptomic and metabolomic datasets revealed strong functional associations. In the LCC group, 97 DEGs and differential metabolites were mapped to the same pathways, while 86 such overlaps were identified in the HCC group (Fig. S2C–D). The overlapping pathways were largely consistent across treatments and included ABC transporters, arginine and proline metabolism, protein digestion and absorption, and bile secretion (Fig. 6F).

### 3.5 Dietary lemongrass improves immune function and intestinal metabolism in piglets

Inflammatory cytokine levels were measured to further investigate immune responses. Serum concentrations of IFN-γ (*P* < 0.001), TNF-α (*P* < 0.001), IL-1β (*P* < 0.001), and IL-6 (*P* = 0.003) were lower in piglets from both lemongrass-supplemented groups compared with controls (Table 4). Ileal cytokine levels were also reduced in the HCC group, and notably, the LCC group exhibited even lower cytokine levels than the HCC group, suggesting stronger anti-inflammatory effects at the lower supplementation level (Table 4). Given that multi-omics analysis indicated modulation of intestinal lipid and protein metabolism, the levels of FATP and FABP, as well as trypsin and lipase activities were further measured. Lemongrass supplementation increased FATP (*P* = 0.001) and FABP (*P* = 0.004) expression, along with trypsin (*P* = 0.002) and lipase (*P* = 0.004) activities in the ileum (Table 5).

**Table 4:** Effects of lemongrass on inflammatory cytokines in serum and ileum of weaned piglets.

Items	Groups^1^			SEM	*P-*value		
	CON	LCC	HCC		Treatment	Linear	Quadratic
**Serum, pg/mL**							
IFN-γ	15.90^a^	10.30^b^	12.39^b^	0.643	<0.001	0.001	<0.001
TNF-α	37.50^a^	22.55^b^	28.32^b^	2.283	<0.001	0.011	0.002
IL-1β	509.60^a^	419.82^b^	384.99^b^	16.533	<0.001	<0.001	0.192
IL-6	543.21^a^	398.59^b^	410.70^b^	27.452	0.003	0.005	0.024
**Ileum, pg/g**							
IFN-γ	78.64^a^	51.34^c^	65.79^b^	3.025	<0.001	0.008	<0.001
TNF-α	391.26^a^	255.53^c^	366.22^b^	8.732	<0.001	0.058	<0.001
IL-1β	1449.02^a^	904.25^c^	1269.87^b^	37.214	<0.001	0.003	<0.001
IL-6	1407.67^a^	807.00^c^	1207.46^b^	48.117	<0.001	0.009	<0.001

SEM = standard error of the mean; IFN-γ = interferon-gamma; TNF-α = tumor necrosis factor-alpha; IL-1β = interleukin-1 beta; IL-6 = interleukin-6.

Means with different superscripts within the same row differ significantly (*P* < 0.05).

1 CON, the basal diet group; LCC, 0.1 % lemongrass supplementation in the basal diet group; HCC, 0.5 % lemongrass supplementation in the basal diet group.

**Table 5:** Effects of lemongrass on lipid metabolism of weaned piglets.

Items	Groups^1^		SEM	*P*-value
	CON	LCC		
**Ileum**				
Lipase, U/g	22.03^b^	26.84^a^	0.794	0.004
Trypsin, U/g	0.99^b^	1.39^a^	0.074	0.002
FATP, μg/g	0.68^b^	1.18^a^	0.081	0.004
FABP, μg/g	118.14^b^	173.14^a^	5.835	0.001
**Jejunum**				
Lipase, U/g	15.60^b^	20.67^a^	0.876	0.007
Trypsin, U/g	1.01^b^	1.43^a^	0.046	<0.001

SEM = standard error of the mean; FATP = fatty acid transport protein; FABP = fatty acid binding protein.

Means with different superscripts within the same row differ significantly (*P* < 0.05).

1 CON, the basal diet group; LCC, 0.1 % lemongrass supplementation in the basal diet group.

## 4 Discussion

Lemongrass possesses antioxidant, anti-inflammatory, and immunoregulatory properties. In poultry and ruminants, dietary lemongrass has been shown to enhance growth performance and nutrient digestibility (Tiwari et al., 2019; Wanapat et al., 2008, 2013). Citral, one of its major constituents, regulates cytokine secretion in intestinal epithelial cells (Guo et al., 2022) and modulates immune responses in macrophages (Sforcin et al., 2009). However, its potential applications and effects in weaned piglets have not been previously explored. This study demonstrated that dietary lemongrass improved growth performance, intestinal function, inflammatory responses, and gut microbiota in weaned piglets. Multi-omics analyses further indicated that these benefits may be mediated through enhanced nutrient digestion and absorption.

Gut microbiota plays a central role in mediating the beneficial effects of dietary bioactive components (Gong et al., 2020). In this study, lemongrass supplementation increased the relative abundances of *L. reuteri* and *W. paramesenteroides*, both of which promote gut health through lactic acid production, thereby lowering luminal pH and inhibiting pathogenic bacteria (Alagawany et al., 2021). Notably, *L. reuteri* has been reported to enhance intestinal barrier function by upregulating tight junction proteins (Gao et al., 2022), which is consistent with our observation of increased claudin expression following lemongrass supplementation. Compared with supplementation of *L. reuteri* alone, lemongrass produced more pronounced improvements in growth performance (Dell’Anno et al., 2021). The results also showed an increase in *Streptococcus hyointestinalis*, which links to improved host metabolism and immune function (Lee et al., 2020), as well as an enrichment of *K. slooffiae*, a core fungus in the porcine gut that reduces lysine succinylation and promotes glycolysis in intestinal epithelial cells (Hu et al., 2023). These shifts are likely attributable to bioactive compounds such as citral and geraniol, which selectively promote *Lactobacillus* growth while inhibiting *E. coli* and *Salmonella* (Xu et al., 2024; Yang et al., 2020). Collectively, these results suggest that lemongrass modulates the gut microbiota to support barrier integrity and immune regulation.

Metabolomic profiling revealed that lemongrass supplementation markedly altered ileal metabolites associated with nutrient absorption and immune function. Specifically, lemongrass modulated arachidonic acid metabolism, with increased prostaglandin B1 and thromboxane B2 levels and reduced stearic acid, changes that may help maintain immune homeostasis and alleviate metabolic stress (Cai et al., 2023; Xuan et al., 2023). In amino acid metabolism, lemongrass enhanced aromatic amino acid production, which serves as a precursor for norepinephrine, thyroid hormones, and serotonin-molecules essential for appetite regulation, energy metabolism, protein synthesis, and immune function (Fernstrom and Fernstrom, 2007; Fiore and Murray, 2021; Gong et al., 2020; Seo and Kwon, 2023; Silvano et al., 2021; Zhou et al., 2024). Additionally, these amino acids increase tricarboxylic acid (TCA) cycle intermediates (isocitric acid and citric acid) (Astorga et al., 2022; O'Neill and Hardie, 2013) and metabolites involved in neurotransmitter and glucose metabolism (5-aminovaleric acid, malonic acid, and glutaric acid) (Ahmed et al., 2022; Haberbosch et al., 2023), thereby supporting energy availability and tissue repair. These findings highlight the role of lemongrass-induced metabolic shifts in mediating nutrient absorption and immune function. Because host metabolism is strongly influenced by gut microbiota-derived metabolites (Liu et al., 2025, 2023), this study integrated microbiome and metabolome data. Correlation analyses revealed that *Lactobacillus* was positively associated with metabolites such as N-ε-acetyl-L-lysine (amino acid metabolism) and 13-hydroxyoctadecadienoic acid (linoleic acid metabolism), suggesting that *Lactobacillus* may shape host metabolic pathways by modulating phenylalanine and linoleic acid metabolism (Chen et al., 2023; Lv et al., 2020). These integrative findings provide insights into how lemongrass influences microbiota-metabolite-host interactions to improve immunity and reduce inflammation.

Transcriptomic analysis further supported these observations. Several genes in the NF-κB signaling pathway were downregulated in the ileum following lemongrass supplementation. Geraniol, a major component of lemongrass, is known to inhibit NF-κB activation and suppress inducible nitric oxide synthase (iNOS) and cyclooxygenase-2 expression (Medicherla et al., 2015). Consistently, this study observed reductions in pro-inflammatory cytokines, suggesting that NF-κB signaling is a primary target of lemongrass. Integrated transcriptomic-metabolomic analysis revealed enrichment of DEGs and differential metabolites in pathways related to protein digestion and absorption, arachidonic acid metabolism, and ABC transporters. These findings are in line with increased activities of lipase and trypsin, elevated FATP and FABP expression, and enhanced claudin-mediated paracellular nutrient transport (Ong et al., 2020). To our knowledge, this is the first report to demonstrate that lemongrass promotes nutrient digestion and absorption in weaned piglets, potentially through microbiota-mediated production of short-chain fatty acids (SCFAs) by *L. reuteri* and *W. paramesenteroides* (Liu et al., 2024; Xiao et al., 2023; Zhang et al., 2022). These SCFAs may enhance digestive enzyme activity and nutrient uptake (Li et al 2023.; Xie et al., 2024), consistent with the results of enzymatic measurements. However, the study also has several limitations. The short duration restricted understanding of the long-term effects of lemongrass supplementation on piglet growth and health, and the molecular mechanisms behind the benefits of lemongrass are not fully understood. Thus, future research should focus on the long-term effects and elucidate the underlying signaling pathways and molecular interactions.

## 5 Conclusion

This study demonstrates that dietary lemongrass supplementation enhances growth performance, intestinal function, and immune status in weaned piglets. A 0.1% inclusion level proved more effective than 0.5% in improving final body weight, average daily gain, and anti-inflammatory capacity, suggesting that 0.1% (as-fed basis) is the optimal dose. Multi-omics analyses confirmed that lemongrass increased the relative abundances of *L. reuteri*, *W. paramesenteroides*, and *K. slooffiae*, promoted amino acid and lipid metabolism, and altered ileal gene expression related to nutrient absorption and immune regulation. Overall, these findings identify lemongrass as a promising alternative to antibiotics in livestock production.

## Credit Author Statement

**Jing Liang:** Writing – original draft, Methodology, Formal analysis, Data curation. **Zhenmei Zhong:** Writing – original draft, Software, Resources, Investigation, Conceptualization. **Aiyang Wang:** Writing – review & editing, Methodology, Investigation, Data curation. **Yulong Yin:** Writing – review & editing, Funding acquisition. **Kaibin Zheng:** Writing – review & editing, Funding acquisition, Conceptualization. **Xihong Zhou:** Writing – review & editing, Funding acquisition, Conceptualization.

## Declaration of conflict of interest

We declare that we have no financial and personal relationships with other people or organizations that can inappropriately influence our work, and there is no professional or other personal interest of any nature or kind in any product, service and/or company that could be construed as influencing the content of this paper.
