# Supplementary material for: Lemongrass (Cymbopogon citratus) supplementation improves growth performance, intestinal function and inflammation status in weaned piglets
Source: Anim Nutr. 2025 Nov 20;24:61–73. doi: 10.1016/j.aninu.2025.09.007 (PMC12828569; doi:10.1016/j.aninu.2025.09.007)
Supplement: Multimedia component 1 [file mmc1.docx]

**Supplementary files**

**Table S1 Components of lemongrass extract.**

| **Name** | **Percentage, %** | **CAS No.** | **Chemical formula** | **Structural formula** |
| --- | --- | --- | --- | --- |
| 5-Hepten-2-one,6-methyl- | 7.5-8.0 | 110-93-0 | C_5_H_14_O | 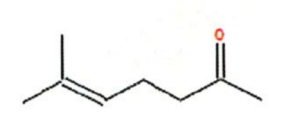 |
| Trans-β-ocimene | 1.5-1.8 | 3779-61-1 | C_10_H_16_ | 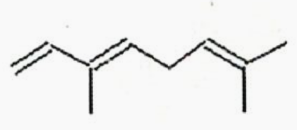 |
| 1,3,6 -Octatrience,3,7-dimethyl-, (z)- | 0.8-1.0 | 3338-55-4 | C_10_H_16_ | 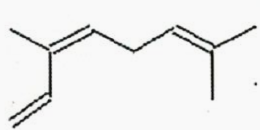 |
| 4-Nonanone | 2 | 4485-09-0 | C_9_H_13_O | 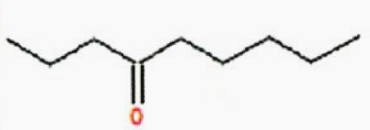 |
| Bicyclo [3,1,1] hept-3-en-2-one, 4,6,6-trimethyl- | 0.5-0.8 | 80-57-9 | C_10_H_14_O | 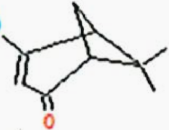 |
| 1.6-octadien-3-ol, 3,7-dimethyl- | 2.0-2.2 | 78-70-6 | C_10_H_15_O | 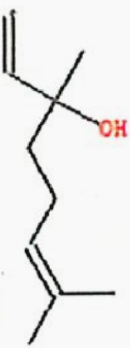 |
| Spiro[bicyclo[3,1,1] heptane-2-2’-oxirane],6, ε-dimethyl- | 0.8-1.0 | 6931-54-0 | C_10_H_16_O | 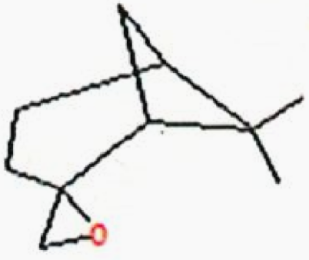 |
| Citronellal | 0.8-1.0 | 106-23-0 | C_10_H_15_O | 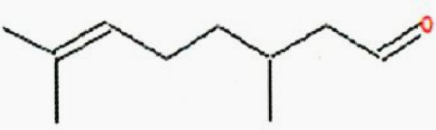 |
| Cis-verbenol | 4.0-4.5 | 1845-30-3 | C_10_H_16_O | 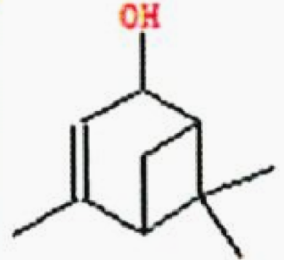 |
| Carane,4,5-epoxy-, trans | 5.0-5.5 | 6909-20-2 | C_10_H_16_O | 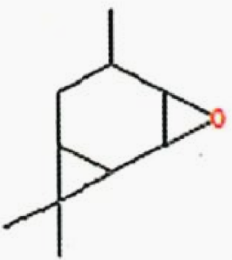 |
| Decanal | 2.0-2.5 | 112-31-2 | C_10_H_20_O | 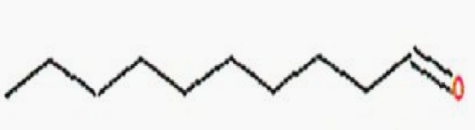 |
| Citral | 53.0-54.0 | 5392-40-5 | C_10_H_16_O | 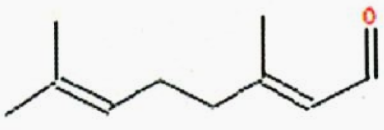 |
| Geraniol | 3.5-4.0 | 106-24-1 | C_10_H_13_O | 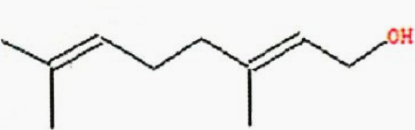 |
| Neric acid | 1.0-1.2 | 4613-38-1 | C_10_H_16_O_2_ | 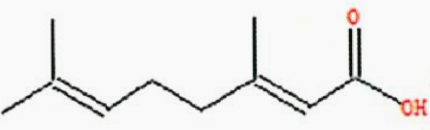 |
| 2.6-Ocradien-1-ol,3,7-dimethyl-, acetate | 1.5-1.8 | 16409-44-2 | C_12_H_20_O_2_ | 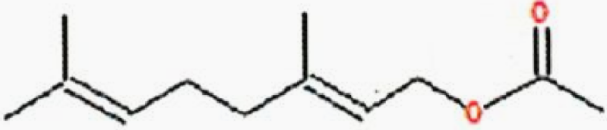 |
| Cyelohexane,10ethenyl-1-methyl-2,4-bis(1-methylethenyl)-,[1s-(1σ，2β，4β]- | 0.3-0.5 | 515-13-9 | C_15_H_24_ | 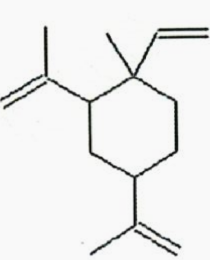 |
| Dodecanal | 0.2-0.4 | 112-54-9 | C_12_H_24_O | 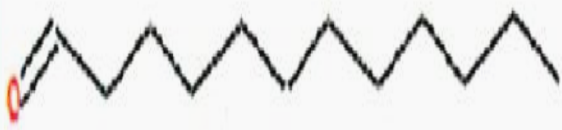 |
| Caryophyllene | 3.5-4.0 | 87-44-5 | C_15_H_24_ | 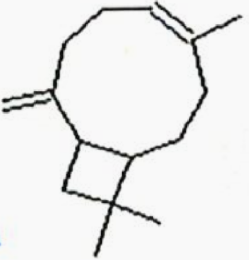 |
| Bicyclo [3,1,1] hept-2-ene,2,σ-dimethyl-6-(4-methyl-pentenyl)- | 1.5-1.8 | 17699-05-7 | C_15_H_24_ | 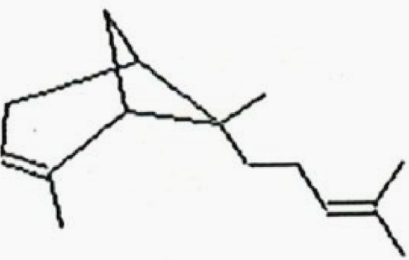 |
| Trans-Isoeugenol | 0.7-0.9 | 5932-68-3 | C_10_H_12_O_2_ | 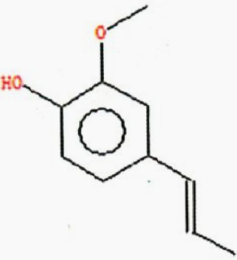 |
| Humulene | 0.8-1.0 | 6753-98-6 | C_15_H_24_ | 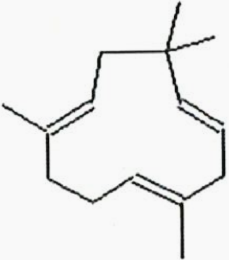 |
| Caryophyllene oxide | 1.5-1.8 | 1139-30-6 | C_15_H_24_O | 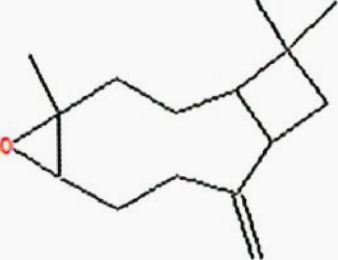 |
| 3,7,11,15-Tetramethyl-2-hexadecen-l-ol | 0.3-0.5 | 102608-53-7 | C_20_H_40_O | 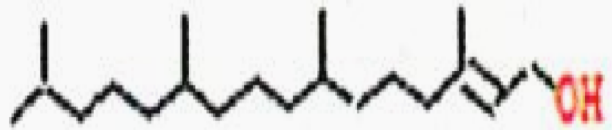 |

**Table S2 Effects of lemongrass on serum indices of weaned piglets.**

| Item | Groups | | | SEM | *P*-value | | |
| --- | --- | --- | --- | --- | --- | --- | --- |
|  | CONT | LCCS | HCCS |  | Treatment | Linear | Quadratic |
| TP, g/L | 56.07 | 52 | 55.33 | 3.278 | 0.652 | 0.874 | 0.369 |
| BUN, mmoL/L | 2.60^b^ | 3.30^ab^ | 4.66^a^ | 0.413 | 0.043 | 0.014 | 0.494 |
| TG, mmoL/L | 0.76^b^ | 1.56^a^ | 1.65^a^ | 0.451 | 0.015 | 0.005 | 0.559 |
| CHOL, mmoL/L | 2.61^b^ | 2.88^ab^ | 3.22^a^ | 0.214 | 0.037 | 0.042 | 0.895 |

SEM = standard error of the mean; TP = total protein; BUN = blood urea nitrogen; TG = triglycerides; CHOL = cholesterol.

Means with different superscripts within the same row differ significantly (*P* < 0.05).

^1^CON = the basal diet group; LCC = 0.1% lemongrass supplementation in the basal diet group.


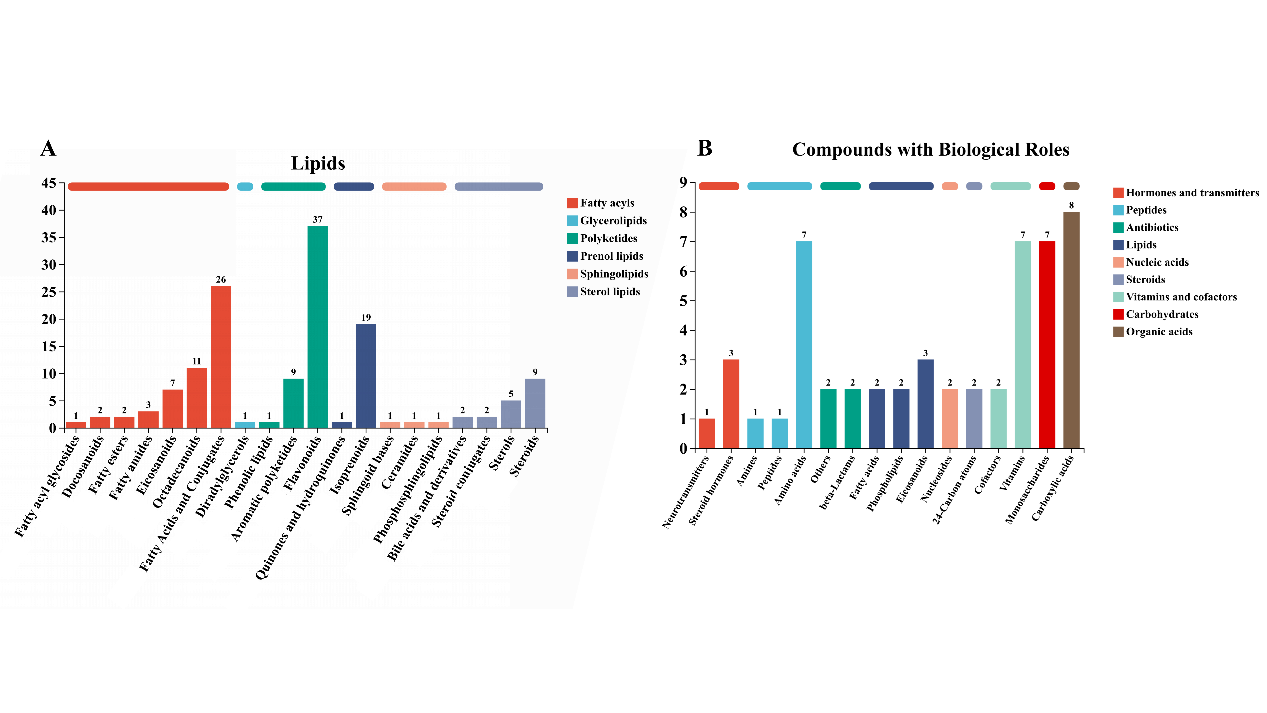


Fig. S1. Kyoto Encyclopedia of Genes and Genomes (KEGG) compound classification and statistical analysis of the differential compounds. (A) KEGG enrichment analysis of lipid-associated compounds. (B) KEGG enrichment analysis of compounds with biological functions.


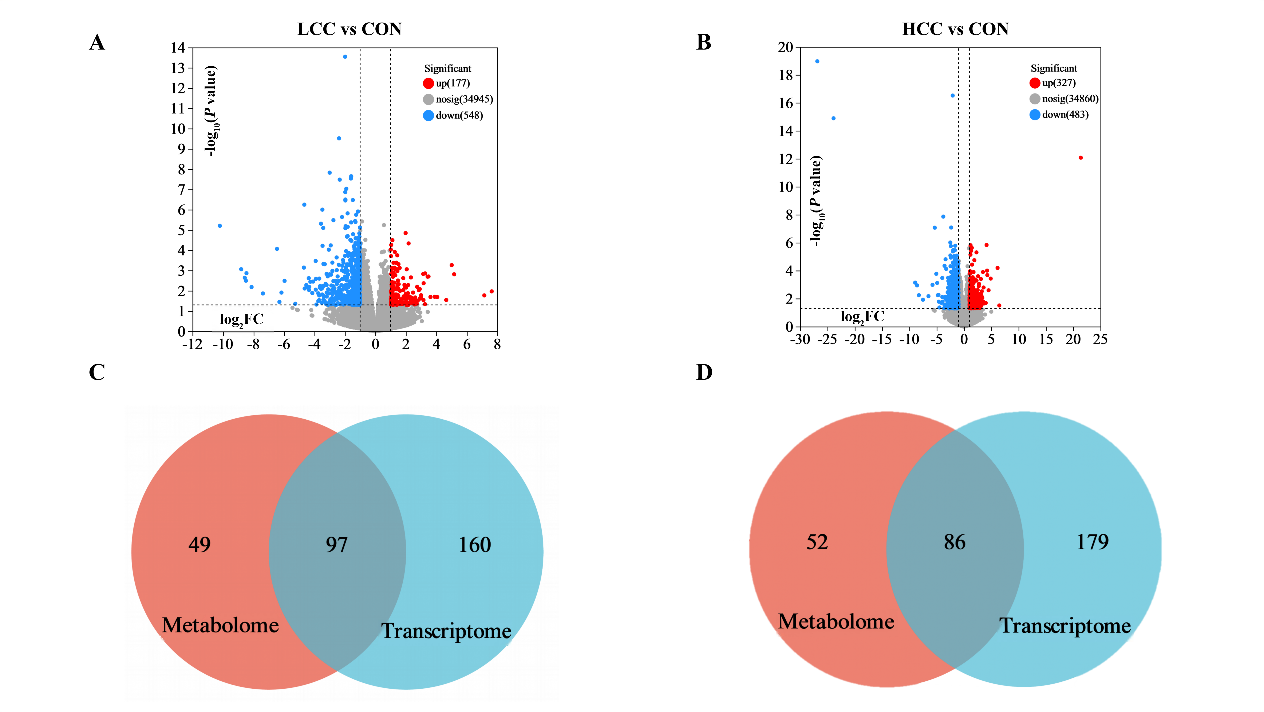


Fig. S2. The volcano plot of differentially expressed genes (DEGs) and Venn plot between DEGs and differential metabolites (DMs). Dietary treatments were as follows: CON (basal diet), LCC (basal diet + 0.1% lemongrass), and HCC (basal diet + 0.5% lemongrass). (A) Volcano plot of DEGs between the CON and LCC groups. (B) Volcano plot of DEGs between the CON and HCC groups. (C) Venn plot showing the overlap between DEGs and DMs in the CON and LCC groups. (D) Venn plot showing the overlap between DEGs and DMs in the CON and HCC groups. FC = fold change.
